# Multimodal therapy in the management of lacrimal gland adenoid cystic carcinoma

**DOI:** 10.1186/s12886-019-1110-5

**Published:** 2019-06-08

**Authors:** Jie Yang, Chuandi Zhou, Yefei Wang, Xianqun Fan, Renbing Jia

**Affiliations:** 1 Department of Ophthalmology, Ninth People’s Hospital, Shanghai Jiao Tong University School of Medicine, 639 Zhi Zao Ju Road, Shanghai, 200011 China; 2 Shanghai Key Laboratory of Orbital Diseases and Ocular Oncology, Shanghai, China

**Keywords:** Lacrimal gland adenoid cystic carcinoma, Eye-sparing surgery, Local recurrence, Metastasis, Tumor-related mortality, 8th American joint committee on Cancer

## Abstract

**Background:**

To evaluate the prognosis of Chinese patients with lacrimal gland adenoid cystic carcinoma treated with eye-sparing surgery and adjuvant multimodal therapy.

**Methods:**

The study included 24 consecutive patients with lacrimal gland adenoid cystic carcinoma treated at the Ninth People’s Hospital of Shanghai from May 2008 to September 2017. All patients underwent eye-sparing surgical tumor resection and 20 (83.3%) of the 24 patients in the cohort received postoperative RT. Eight (41.7%) patients in the cohort received chemotherapy. Each patient’s medical records were reviewed.

**Results:**

The study included 13 male and 11 female patients. The median follow-up time after surgery was 33.5 months. Fifteen (62.5%) patients experienced local recurrence. The 1-, 3-, and 5-year recurrence rates were 27.9, 60.0, and 80.0%, respectively. Eleven (45.8%) patients developed metastasis. The 1-, 3-, and 5-year metastasis rates were 8.7, 48.5, and 66.9%, respectively. Eight (33.3%) patients died of lacrimal gland adenoid cystic carcinoma, with a median survival duration of 34.0 months. The 1-, 3-, and 5-year tumor-related mortality was 4.5, 28.1, and 58.0%, respectively. More advanced T stage (≥ T3a) was a risk factor for local recurrence (hazard ratio [HR]: 5.374, *P* = 0.02), distant metastasis (HR: 8.585, *P* < 0.01), and tumor-related survival (HR: 9.654, *P* < 0.01).

**Conclusions:**

Eye-sparing tumor resection protocol followed by adjuvant therapy seems to be associated with high rates of local recurrence, metastases and death. In addition, greater attention should be paid to patients with lacrimal gland adenoid cystic carcinoma with ≥ T3a tumors.

## Background

Lacrimal gland adenoid cystic carcinoma (LGACC) accounts for 25–40% of all epithelial tumors of the lacrimal gland [1]. In China, adenoid cystic carcinoma (20.0%) is one of the most common epithelial tumors of the lacrimal gland [2]. In addition, Lacrimal gland adenoid cystic carcinoma has an aggressive behavior and is associated with significant morbidity and mortality [3, 4].

In the literature, the overall tumor-related mortality of LGACC is 10–87% [5–13]. LGACC exhibits local recurrence and unfavorable outcomes even when managed with aggressive local treatment [7, 14–20].

The different therapeutic options for LGACC have be described by institution. The traditional surgical treatment is orbital exenteration. Orbital exenteration followed by radiotherapy has been widely used for lacrimal gland carcinoma without distant metastasis [21]. For adjuvant radiation therapy, various types have been reported, including external-beam radiation therapy, proton therapy, and brachytherapy [6–8, 22–26]. However, in recent articles, radical surgical treatment did not improve the overall survival outcomes of patients with LGACC [5, 27]. In addition, studies have demonstrated that the quality of life of patients who underwent orbital exenteration is markedly reduced [28]. In 2013, one excellent study by Tse. and colleagues clarified neoadjuvant intra-arterial cytoreductive chemotherapy (IACC) may improve overall survival and decrease disease recurrence. Furthermore, the chemotoxicity complication rate is limited and manageable [7].

**Fig. 1:**
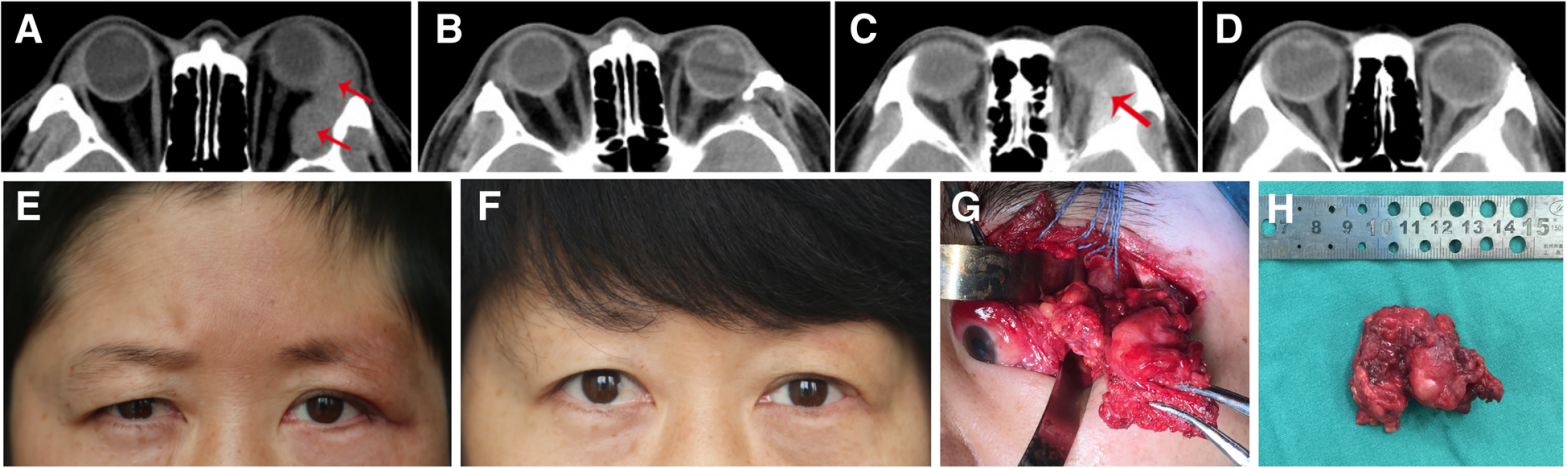
CT scan of Case 9, who had adenoid cystic carcinoma of the lacrimal gland, and axial computed tomographic scan after surgical resection (**a**, **b**). Photos and CT scan of Case 20, who had adenoid cystic carcinoma of the lacrimal gland, and axial computed tomographic scan after surgical resection (**c**, **d**, **e**, **f**). Intraoperative photograph for eye-sparing tumor excision (**g**). Excised tumor (**h**)

In the past decade, our institution has performed eye-sparing surgery in patients with lacrimal gland adenoid cystic carcinoma (Fig. 1). In this report, we evaluated the outcomes, including local recurrence, distant metastases, and survival after eye-sparing surgery, for LGACC in Chinese patients and explore the prognostic factors for local recurrence and metastasis.

## Methods

### Patients

All consecutive patients of LGACC who were treated with eye sparing surgery between May 2008 and September 2017 at the Ninth People’s Hospital of Shanghai were retrospectively reviewed. The therapy sequence is surgery, then RT and intravenous chemotherapy last. The recurrence cases were given the same modality of treatment. The exclusion criteria were as follows: (1) incomplete available clinical data and (2) a follow-up period of < 3 months. We contacted all patients who were eligible for inclusion or their relatives, which were legal guardians. We explained the purpose of our study, and the patients all volunteered to participate in this study. Informed consent in verbal form was obtained from all patients or their relatives at the follow-up visit. This study adhered to the tenets of the Declaration of Helsinki and was approved by the Shanghai JiaoTong University research ethics committee.

### Data collection

The patients’ clinical information included age, sex, duration of symptoms, greatest basal diameter of tumor, presence of perineural invasion, findings on imaging studies (magnetic resonance imaging (MRI) or computed tomography (CT)), the presence of adjuvant treatments and 8th AJCC stage at presentation to our hospital. The main outcome measures were local recurrence, distant metastasis, death due to disease or unrelated causes, and the time from surgery of each event. The local recurrence and distant metastasis confirmed by MRI or CT scanning.

### Statistical analysis

All data were processed using the Statistical Package for the Social Sciences software package, version 23.0 (SPSS, Chicago, IL). First, we used univariate Cox proportional hazards regression to identify possible factors associated with recurrence, metastasis, and tumor-related death. Regression coefficients and hazard ratios (HRs) with 95% confidence intervals were calculated. The Kaplan-Meier method was used to analyze the local recurrence, metastasis, and tumor-related death rates. In addition, the log-rank test was used to compare the overall survival of patients with<T3a and ≥ T3a primary tumors. All tests were 2-sided, and a *P* value of < 0.05 was considered statistically significant.

## Results

### Clinical data

A total of 24 patients were recruited in this study; 13 (54.2%) were male, and 11 (45.8%) were female (Table 1). The age of the patients ranged from 32.0 to 83.0 years, with a median of 54.0 years. Treatment before referral to our hospital was noted in 5 (20.8%) patients. And they were received a biopsy and eye-sparing surgical tumor resection. Primary symptoms included exophthalmos (7, 29.2%), orbital pruritus (1, 4.2%), a mass noted on imaging studies (5, 20.8%), eyeball displacement (2, 8.3%), tearing (1, 4.2%), ptosis of the upper eyelid (1, 4.2%), pain (5, 20.8%), and decreased vision (2, 8.3%). The median duration of symptoms was 6.0 months (range: 1.0–120.0 months). The median tumor dimension was 26.5 mm (range: 12.0–55.0 mm). At the time of initial diagnosis, one (4.2%) patient (case 10) had distant metastasis in the brain, confirmed by CT scanning.

**Table 1:** Characteristics of patients with lacrimal gland adenoid cystic carcinoma

Case No.	Age (years)/Gender/Affected side	Duration of symptom (months)	Greatest dimension (mm)	PNI	8th AJCC stage	Treatment	LR/Met	Outcome
1	58/F/L	6	19	+	T1bN0M0	E + RT	no	NED
2	34/M/R	2	45	+	T3bN0M0	E + RT	LR/brain	DOD
3	56/M/L	6	20	–	T1aN0M0	E + RT + CT	no	NED
4	58/M/R	1	38	–	T2aN0M0	E + RT	no	NED
5	58/M/L	6	30	+	T2bN0M0	E + RT + CT	LR	NED
6	45/F/R	2	30	+	T2bN0M0	E + RT + CT	LR	NED
7	83/M/L	96	35	–	T2aN0M0	E + RT	LR/lung	DOD
8	59/F/L	8	26	+	T2bN0M0	E + RT	no	NED
9	38/M/L	6	38	+	T2bN0M0	E	LR	NED
10	54/F/L	12	55	+	T4cN0M1	E	LR/brain	DOD
11	51/F/L	12	24	+	T2bN0M0	E + RT + CT	LR/lung, brain	AWD
12	45/M/R	24	27	–	T2aN0M0	E	LR	NED
13	66/F/L	120	23	–	T2aN0M0	E + RT	no	NED
14	38/F/R	3	12	–	T1aN0M0	E + RT	LR	NED
15	44/F/R	24	20	–	T1aN0M0	E + RT + CT	lung	DOD
16	71/M/L	6	13	–	T1aN0M0	E + RT + CT	LR/liver	AWD
17	32/M/R	2	26	–	T2aN0M0	E + RT + CT	LR/lung, brain	DOD
18	52/M/R	2	45	+	T3bN0M0	E + RT + CT	LR/LN, brain	DOD
19	72/F/L	72	20	–	T1aN0M0	E + RT	LR/nose	AWD
20	36/F/L	2	22	+	T2bN0M0	E + RT + CT	no	NED
21	61/F/R	24	45	+	T3bN0M0	E + RT + CT	LR/brain	DOD
22	54/M/L	6	31	+	T2bN0M0	E + RT	no	NED
23	49/M/L	6	35	+	T2bN0M0	E + RT	LR/brain	DOD
24	63/M/L	24	20	–	T1aN0M0	E	no	NED

*F* female, *M* male, *PNI* perineural invasion *E* excision, *RT* postoperative radiation therapy, *CT* Chemotherapy, *LR* local recurrence, *Met* distant metastases, *LN* lymph node, *DOD* died of disease, *NED* no evidence of disease, *AWD* alive with metastatic disease

**Fig. 2:**
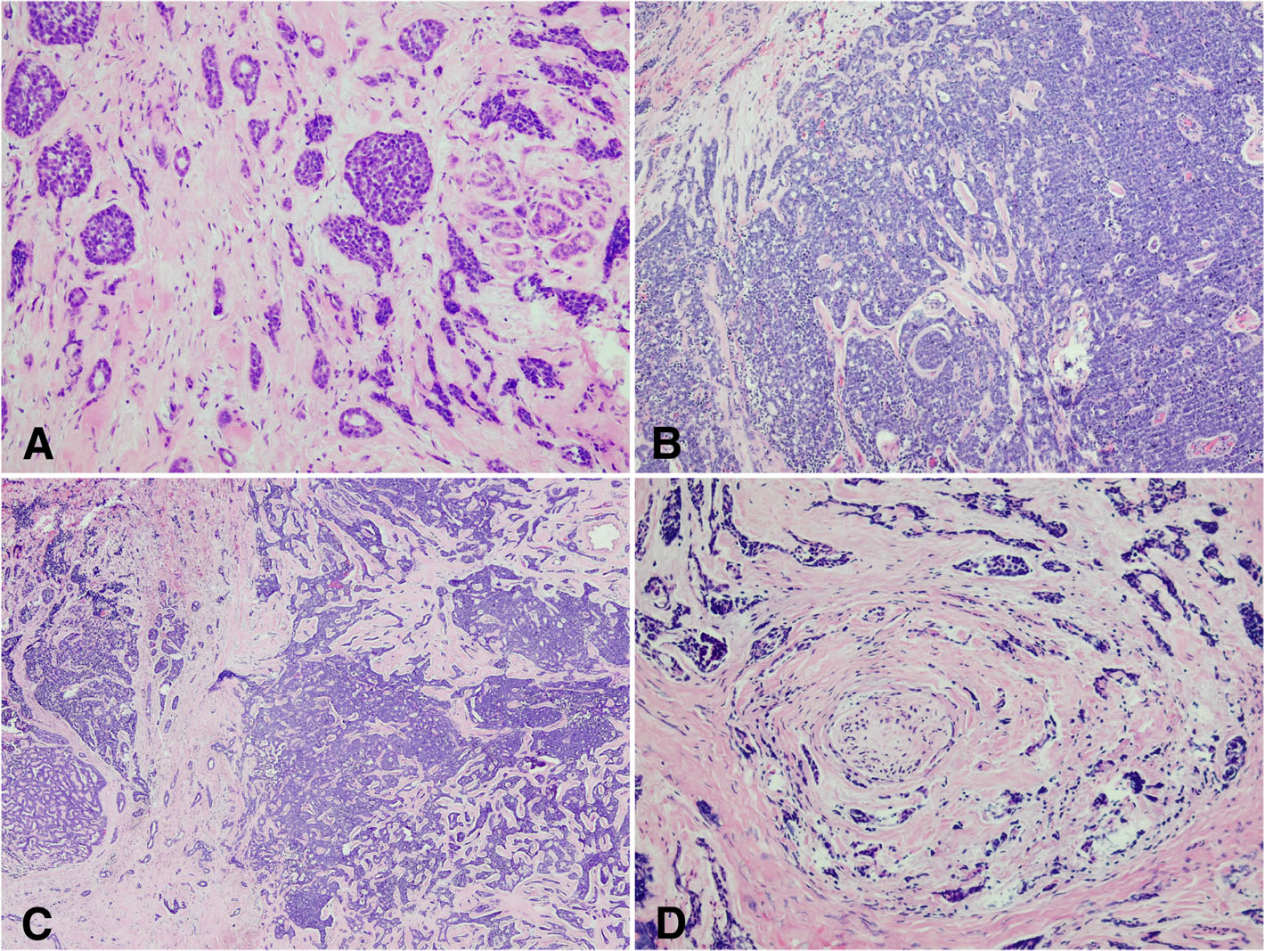
Histopathologic features of adenoid cystic carcinoma of the lacrimal gland. (**a**, **b**). Histologic section of mixed pattern of adenoid cystic carcinoma of lacrimal gland (**c**). Perineural invasion (**d**)

The distribution of tumor histologic subtypes of 21 (87.5%) patients were collected (Fig. 2). It is summarized as follows: predominantly basaloid (solid), 2 (9.5%) patients; predominantly cribriform, 13 (61.9%) patients; mixed, 6 (28.6%) patients; and tubular, 0 patient. Additionally, 13 (51.2%) patients had perineural invasion based on histologic evidence.

Preoperative imaging findings were available for 20 (83.3%) patients. Four other patients received CT scans in the outer hospital and data failure. Of these, 14 (70.0%) patients had CT or MRI evidence of bone involvement.

### American joint committee on Cancer classification at initial diagnosis

The 8th AJCC classification for LGACC at initial diagnosis for the 24 patients was as follows: T1aN0M0, 6 (25.0%) patients; T1bN0M0, 1 (4.2%) patient; T2aN0M0, 5 (20.9%) patients; T2bN0M0, 8 (33.3%) patients; T3bN0M0, 3 (12.5%) patients; and T4cN0M1, 1 (4.2%) patient (Table 1). Four (16.7%) of the 24 patients had ≥ T3a tumors at presentation.

### Treatment modalities

All (100.0%) patients received eye-preserving surgery. And if the patients’ preoperative imaging suggested bony involvement, the affected orbital bone will be removed. If not, performed gross total resection of the lacrimal gland mass. Overall, 20 (83.3%) of the 24 patients in the cohort received postoperative RT, and no one discontinued treatment because of complications. In addition, 4 patients did not receive postoperative adjuvant therapy because they refused. Eight (41.7%) patients in the cohort received chemotherapy.

### Outcome

The median follow-up period was 33.5 months (range: 3.0–113.0 months). During the follow-up, 15 (62.5%) patients had local recurrence; among them, eight (33.3%) patients experienced recurrence within the first year after initial diagnosis. Five (20.8%) patients had more than two recurrences. The median duration to the initial recurrence was 16.0 months (mean: 23.3 ± 16.1 months; range: 4.0–58.0 months). By Kaplan-Meier survival estimates, the 1-, 3-, and 5-year recurrence rates were 27.9, 60.0, and 80.0%, respectively.

In this study, 11 (45.8%) patients developed metastasis. The initial metastasis locations included the cervical lymph nodes (1, 5.2%), brain (7, 29.2%), liver (1, 5.2%), lung (4, 16.7%), and nose (1, 5.2%). Among these patients, three (12.5%) presented with metastases involving multiple sites. The median duration between the initial diagnosis and first metastasis was 25.0 months (mean: 28.4 ± 17.1 months; range: 4.0–68.0 months). By Kaplan-Meier survival estimates, the 1-, 3-, and 5-year metastasis rates were 8.7, 48.5, and 66.9%, respectively.

During follow-up, 8 (33.3%) patients died of distant metastasis from LGACC, with a median survival time of 34.0 months (mean: 35.6 ± 21.2 months; range: 5.0–74.0 months). By Kaplan-Meier survival estimates, the 1-, 3-, and 5-year tumor-related mortality was 4.5, 28.1, and 58.0%, respectively.

Univariate Cox analysis (Table 2) indicated that tumor diameter is a risk factor for recurrence (HR: 4.573, *P* = 0.032), metastasis (HR: 4.024, *P* = 0.045), and tumor-related survival (HR: 8.125, *P* < 0.01).

**Table 2:** Univariate analyses of clinical characteristics for tumor-related death, metastasis, and local recurrence in Chinese patients with lacrimal gland adenoid cystic carcinoma

Characteristics	Total	Recurrence (+)	Recurrence (−)	*P*	Metastasis (+)	Metastasis (−)	*P*	Tumor-related death (+)	Tumor-related death (−)	*P*
Sex	24			0.668			0.818			0.540
Male, no. (%)	13 (54.2)	9 (60.0)	4 (44.4)		6 (54.5)	7 (53.8)		5 (62.5)	8 (50.0)	
Female, no. (%)	11 (45.8)	6 (40.0)	5 (55.6)		5 (45.5)	6 (41.2)		3 (37.5)	8 (50.0)	
Age y	53.2 ± 12.9	52.2 ± 14.8	54.9 ± 9.4	0.901	54.8 ± 15.9	51.9 ± 10.2	0.407	51.1 ± 16.2	54.3 ± 11.3	0.990
Affected Side	24			0.740			0.979			0.398
Right, no. (%)	10 (41.7)	7 (46.7)	3 (33.3)		5 (45.5)	5 (38.5)		5 (62.5)	5 (31.3)	
Lift, no. (%)	14 (58.3)	8 (53.3)	6 (66.7)		6 (54.5)	8 (61.5)		3 (37.5)	11 (68.7)	
RT				0.319			0.626			0.894
Yes, no. (%)	20 (83.3)	12 (80.0)	8 (88.9)		10 (90.9)	10 (76.9)		7 (87.5)	13 (81.3)	
No, no. (%)	4 (16.7)	3 (20.0)	1 (11.1)		1 (9.1)	3 (23.1)		1 (12.5)	3 (18.7)	
CT				0.326			0.193			0.299
Yes, no. (%)	10	7 (46.7)	3 (33.3)		6 (54.5)	4 (30.8)		4 (50.0)	6 (37.5)	
No, no. (%)	14	8 (53.3)	6 (66.7)		5 (45.5)	9 (69.2)		4 (50.0)	10 (62.5)	
diameter mm	29.1 ± 11.0	32.0 ± 12.3	24.3 ± 6.4	0.032^*^	33.0 ± 13.4	25.8 ± 7.6	0.045^*^	38.3 ± 11.5	24.6 ± 7.6	0.004^*^
PNI				0.234			0.890			0.345
Yes, no. (%)	13	9 (60.0)	4 (44.4)		6 (54.5)	7 (53.8)		5 (62.5)	8 (50.0)	
No, no. (%)	11	6 (40.0)	5 (55.6)		5 (45.5)	6 (41.2)		3 (37.5)	8 (50.0)	

PNI = perineural invasion; RT = postoperative radiation therapy; CT = Chemotherapy

According to the 8th edition of the AJCC classification for LGACC, we stratified all patients into 2 groups by their clinicopathologic characteristics. In our study, all patients with ≥ T3a tumors experienced local recurrence, distant metastasis and died of the disease. In contrast, 11 (55.5%) of 20 (83.3%) patients with < T3a tumors experienced local recurrence, 7 (35.0%) experienced distant metastasis, and 4 (20.0%) died of the disease. Thus, the incidence of poor outcomes is much higher in patients with ≥ T3a tumors than in patients with < T3a tumors.

**Fig. 3:**
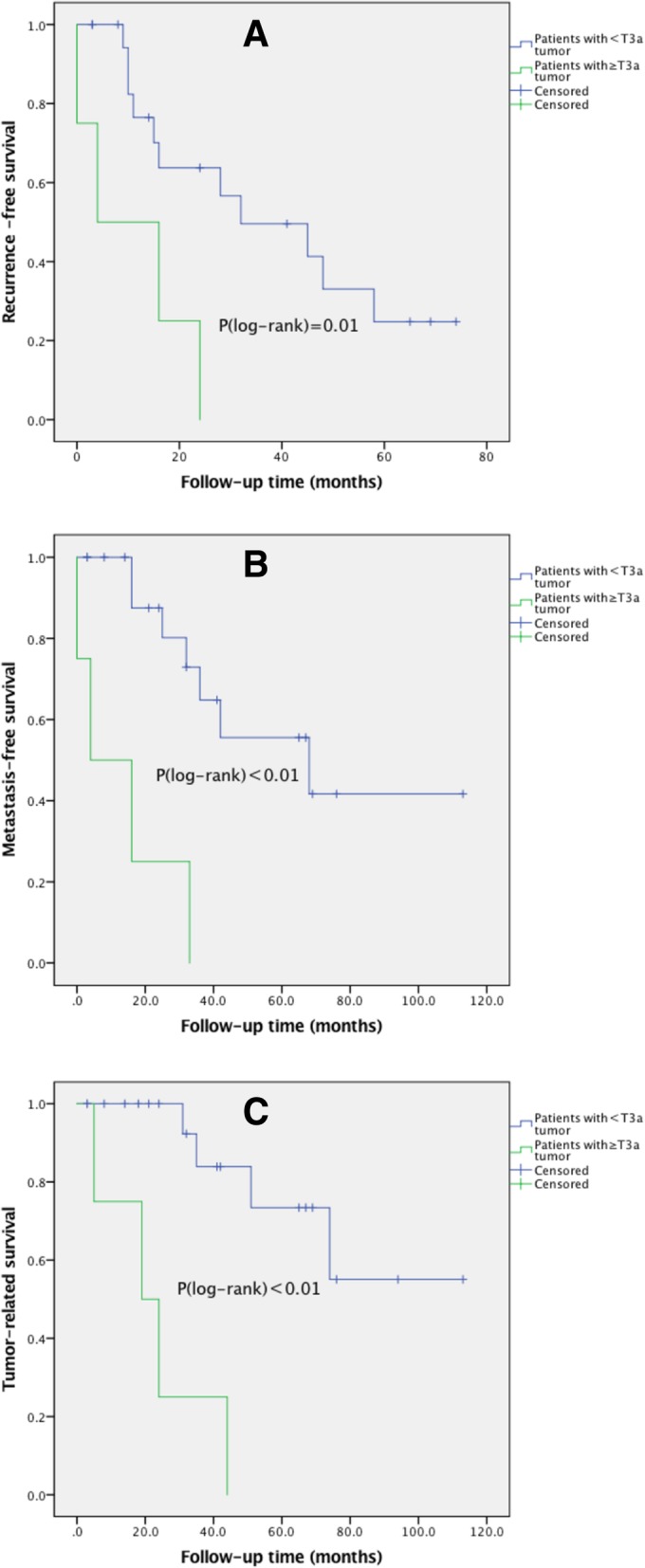
The presence of T3a stage and prognosis in Chinese patients with lacrimal gland adenoid cystic carcinoma. Significantly lower recurrence-free (**a**), metastasis-free (**b**) and tumor-related (**c**) survival rates were observed in patients with < T3a and ≥ T3a tumors according to the 8th AJCC classification (all *P* < 0.05)

The Kaplan-Meier recurrence-free survival, metastasis-free survival and tumor-related survival curves of patients with ≥ T3a tumors and < T3a tumors are shown in Fig. 3. The rates of local recurrence, metastasis and death were significantly different between patients with ≥ T3a tumors and patients with < T3a tumors (all *P* < 0.05).

## Discussion

Based on our observation, ≥ T3a tumors according to the 8th AJCC classification at initial diagnosis were related to poor outcomes. Univariate Cox analysis showed an association with recurrence, metastasis and tumor-related survival. These results are consistent with the findings of Ahmad et al. The authors indicated that ≥ T3 tumors at initial diagnosis were related to poor outcomes. Overall survival was worse, and time to metastasis was significantly shorter for patients with ≥ T3 tumors than for patients with < T3 tumors [14].

Based on published cases in the rest of the world, the exhibited recurrence rates are America (9.1% [5], 0.0% [6], 15.8% [7], 16.7% [8], 44.6% [9], 35.0% [10], and 16.7% [11]), India (10.0% [12]), and Japan (50.0% [13]); and tumor-related death rates are America (0.0% [5], 71.4% [6], 84.2% [7], 38.9% [8], 34.4% [9], 65.0% [10], and 44.4% [11]), and Japan (30.0% [13]). Our findings indicated that 15 (62.5%) patients experienced local recurrence, and 8 (33.3%) patients died of distant metastasis. Based on worldwide data, the recurrence rate in our study was in the higher end of the range, and the tumor-related death rate was comparable to international reports. The reasons were possibly as follows: 1. the histologic subtypes are different. Prior studies showed the basaloid histologic subtype has been suggested to be related with poor outcomes in patients, and in this study, the patients had no basaloid histologic subtype; 2. different doctors’ surgical techniques and methods are different.

Worldwide, there is no agreement regarding the standard for optimal surgery for LGACC [10, 14, 23]. Developing effective treatments for LGACC is especially difficult for at least the following reasons: 1. the incidence of LGACC is very low [29]; 2. because of the complicated structure of the eye, development of new methods has been particularly challenging for ophthalmologists; and 3. the time between initial diagnosis and death is long [30].

Appropriate local treatment for LGACC remains controversial [6, 8]. Historically, orbital exenteration has been the most common surgery for lacrimal gland carcinoma [5, 30]. However, the radical surgical approach did not seem to reduce the patient’s rates of recurrence, metastasis and mortality [6, 10] but reduced the patient’s quality of life because of functional disability and disfigurement [30, 31]. Esmaeli et al. reported favorable outcomes of eye-sparing tumor excision combined with adjuvant RT or chemo-radiotherapy for 11 patients with lacrimal gland carcinoma. Seven patients were diagnosed with LGACC. In their study, all 11 patients were free of disease at last contact [5]. Recently, Jisang Han’s team presented their results. Nine of 10 patients in their cohort did not experience any local recurrence, and the remaining patient with local recurrence was successfully treated with orbital exenteration. The authors also think that eye-sparing surgery followed by adjuvant RT should be considered [31]. And also, eye-sparing surgery with high-dose proton beam radiation provides a variable period of useful vision, and has a nice long-term survival [32]. However, our patients are related to high rates of local recurrence, metastases and death. This may be should consider perineural invasion and subclinical metastases.

Differences were determined based on whether the patients received postoperative adjuvant therapy. Ahmad et al. observed that among patients with ≥ T3 tumors, the risk of local recurrence was increased in patients who did not receive postoperative RT [14]. According to our findings, compared with 12 (60.0%) of 20 patients who were treated with postoperative RT, three (75.0%) of the 4 patients who were not treated with postoperative adjuvant therapy had local recurrence. However, 7 of 10 (70%) patients who received concurrent chemo-radiotherapy, also had local recurrence. Ten patients received postoperative RT only, and 5 (50.0%) had local recurrence. According to these observations, in patients with LGACC, because of eye-sparing surgery, small lesion residue will lead to high recurrence rate, the mortality did not increase.

However, in our study, perineural invasion, which has been previously reported as an independent risk factor [19, 33], had no significant correlation with tumor local recurrence, metastasis or death (*P* > 0.05). The reason may be the relatively small sample size of our study.

While multivariate analysis based on this small cohort would be challenging, obtaining statistically significant conclusions is difficult. However, importantly, this study is the first evaluation comparing detailed clinical characteristics and outcomes in Chinese patients with LGACC and one of largest series of patients with eye-sparing surgery of LGACC reported to date. A previous published report suggested that eye-preserving surgery followed by RT is appropriate only for patients with < T3 tumors, whereas patients with ≥ T3 tumors should be treated with orbital exenteration with bone removal and RT [14]. However, that report was based on the 6th edition of AJCC criteria which was very different from the 8th edition. Therefore, our findings are not comparable to the results observed by Woo et al. [27], Esmaeli et al. [5] and Han et al. [31]. Future studies comparing eye-sparing surgery with orbital exenteration for LGACC are needed to validate the effectiveness and safety of eye-sparing surgery in patients who are treated with adjuvant treatments.

## Conclusions

Eye-sparing tumor resection protocol followed by adjuvant therapy seems to be associated with high rates of local recurrence, metastases and death. In addition, greater attention should be paid to patients with lacrimal gland adenoid cystic carcinoma with ≥ T3a tumors.

## Abbreviations

AJCC: American Joint Committee on Cancer
CT: Chemotherapy
CT: Computed tomography
LGACC: Lacrimal gland adenoid cystic carcinoma
MRI: Magnetic resonance imaging
PNI: Perineural invasion
RT: Postoperative radiation therapy
